# NIAAA 50th Anniversary Festschrift: From the Editor

**DOI:** 10.35946/arcr.v42.1.14

**Published:** 2022-11-17

**Authors:** George F. Koob

**Affiliations:** National Institute on Alcohol Abuse and Alcoholism, Bethesda, Maryland

In 2020, the National Institute on Alcohol Abuse and Alcoholism (NIAAA) celebrated its 50th anniversary. In honor of this important milestone, the Institute organized a 2-day scientific symposium, entitled “Alcohol Across the Lifespan: 50 Years of Evidence-Based Diagnosis, Prevention, and Treatment Research,” that featured presentations by leading researchers who discussed research advances across many domains of alcohol research. The articles in this Festschrift topic series are based on these presentations.

## A Look Back

NIAAA’s 50th anniversary is truly a highlight in the history of public health. More than 5 decades ago, a group of researchers, advocates, and elected officials made a farsighted decision when they pushed for the creation of a federal institution dedicated to research that improves the lives of millions of Americans and their families devastated by alcohol misuse. As a result, on December 31, 1970, President Richard Nixon signed the Comprehensive Alcohol Abuse and Alcoholism Prevention, Treatment, and Rehabilitation Act of 1970.

The Act launched NIAAA, authorizing the new agency to develop and conduct comprehensive health, education, training, research, and planning programs for the prevention and treatment of alcohol-related problems.^1^ The creation of NIAAA set the stage for a trusted, federally funded agency to plan and support advances in a variety of domains, ranging from alcohol’s effects on the developing adolescent brain to alcohol-associated liver disease and from fetal alcohol spectrum disorder to the treatment of alcohol use disorder (AUD).

The Institute also helped change the way we view alcohol misuse and AUD. It provided evidence that AUD is a chronic health condition, not a moral failing. AUD is now conceptualized as a preventable and treatable brain disorder with symptoms that vary across the life span and by individual.

Thanks to innovative research funded by NIAAA, we have a better understanding of how alcohol affects the brain and other organs across the life span. We have also developed evidence-based interventions to prevent and treat alcohol misuse and AUD. Progress has been made in numerous areas, from decreasing underage drinking to understanding fetal alcohol spectrum disorder and to stimulating medications development for treatment of AUD.

The presentations at the 50th Anniversary Symposium represented a small sample of these crucial advances.

## A Look Ahead

NIAAA today is the world’s largest funder of alcohol research, with a mission of improving the diagnosis, prevention, and treatment of AUD and other alcohol-related problems across the life span. With its broad research portfolio, NIAAA’s work focuses on health topics that touch the lives of almost every family and community across the United States.

Yet, despite our progress, many challenges remain. The scope of alcohol misuse and the associated problems place a significant and growing burden on public health and our health care system. Alcohol-related deaths number more than 140,000 per year, making alcohol a leading cause of preventable death in the United States.^2^ Alcohol misuse is also associated with an increased risk of injuries, chronic illnesses such as liver and heart disease, and cancer.^3^ Overall, substantially more individuals suffer from AUD (14,504,000 people, or 5.3% of the population)^4^ than from opioid use disorder (2,060,000 people, or 0.8% of the population).^4^ I often say that “AUD is the addiction that everyone knows about, but nobody wants to talk about.”

A significant challenge is the co-occurrence of alcohol misuse and AUD with other disorders, and how to best help affected individuals recover from both conditions. For example, many individuals with AUD also suffer from other mental health conditions and may use alcohol to cope with these conditions. Additionally, these disorders frequently exacerbate each other. Pain also often co-occurs with alcohol misuse. Although acute alcohol consumption at binge-drinking levels may lead to a temporary reduction of pain, chronic alcohol use and alcohol withdrawal in fact increase pain sensitivity.^5^ Perhaps even more problematic, chronic alcohol use and alcohol withdrawal increase emotional pain, termed hyperkatifeia.^6^ Finally, alcohol misuse, and particularly abstinence after chronic use, can result in persistent sleep problems that promote relapse and thus are a major impediment to recovery from AUD.^7^

In coming years, researchers will need to pay close attention to emerging trends in alcohol use in the U.S. population. Research shows that gender gaps are narrowing for numerous alcohol-related parameters, and that overall prevalence of drinking, prevalence of early-onset drinking, frequency and intensity of drinking, prevalence of AUD, and many negative consequences of alcohol misuse are increasing in women.^8^ Similarly, alcohol use is increasing among adults age 65 and older, and 1 in 10 individuals in this age group engages in binge drinking.^9^ Given that the older population is growing at an unprecedented rate,^10^ this is an important public health concern.

Another major challenge is closing the persistent treatment gap. In 2019, fewer than 8% of people with AUD in the United States received any form of treatment.^4^ Routine health care visits present a unique opportunity for prevention, early intervention, and treatment of AUD, yet many health care providers do not perform alcohol screening, are not aware of evidence-based treatments, or do not know where to refer patients for treatment.^11^ Therefore, it remains essential to improve health care provider training in substance misuse prevention and treatment at all levels and to integrate prevention, early intervention, and treatment into routine health care. To address this need, NIAAA developed the Healthcare Professional’s Core Resource that provides health care providers—from pharmacists, nurse practitioners, physician assistants, clinical psychologists, and primary care physicians to board-certified addiction specialists—all the information they should know about alcohol.

## The 50th Anniversary Scientific Symposium: Festschrift Topic Series

The articles in this Festschrift topic series of *Alcohol Research: Current Reviews* highlight some of the key discoveries made possible over the last 50 years through NIAAA funding to grantees and support to the Institute’s intramural researchers. Epidemiological research has enabled us to track progress and challenges associated with alcohol misuse in the U.S. population overall as well as in various subpopulations. Some of these findings are reviewed in Dr. Keyes’ article, “Age, Period, and Cohort Effects in Alcohol Use in the 20th and 21st Centuries: Implications for the Coming Decades.”^12^

Significant advances also have been made in understanding the genetic basis of AUD and the identification of relevant genes. Dr. Schuckit’s article, “AUD Risk, Diagnoses, and Course in a Prospective Study Across Two Generations: Implications for Prevention,” describes some of these findings, as well as their implications for prevention of alcohol misuse and AUD and for potential precision medicine approaches to the treatment of AUD.^13^

Research also has established that the adolescent brain is uniquely vulnerable to the effects of alcohol. As described by Dr. Tapert and Dr. Eberson-Shumate in the article, “Alcohol and the Adolescent Brain: What We’ve Learned and Where the Data Are Taking Us,” longitudinal studies that assess predictors and consequences of adolescent alcohol consumption continue to inform prevention and treatment strategies aimed at this age group.^14^

Another important topic is recovery from AUD, as many individuals will eventually suffer a relapse to alcohol use, often even after extended periods of abstinence. Dr. Sinha’s article, “Alcohol’s Negative Emotional Side: The Role of Stress Neurobiology in Alcohol Use Disorder,” reviews the current understanding of the role of stress neurobiology in alcohol misuse and its implications for the risk of, and recovery from, AUD.^15^

Decades of research have paved the way for behavioral interventions and medications to help people recover from AUD. Dr. Mason’s article, “Looking Back, Looking Forward: Current Medications and Innovative Potential Medications to Treat Alcohol Use Disorder,” reviews the state of knowledge of the three medications currently approved by the U.S. Food and Drug Administration for the treatment of AUD, as well as introduces other medications with potential for treating AUD that are on the horizon.^16^

The developing fetus is uniquely sensitive to alcohol exposure. Research on understanding how prenatal alcohol exposure affects development as well as on the prevention and mitigation of the effects of prenatal alcohol exposure have been a long-standing research priority for NIAAA since the recognition of fetal alcohol syndrome in the early 1970s. Dr. Charness summarizes research demonstrating advances in our understanding in “Fetal Alcohol Spectrum Disorders: Awareness to Insight in Just 50 Years.”^17^

Finally, Dr. Ramkissoon and Dr. Shah review the current state of knowledge for one of the most common consequences of alcohol misuse, alcohol-associated liver disease, in “Alcohol Use Disorder and Alcohol-Associated Liver Disease.” Alcohol-associated liver disease is a major contributor to alcohol-related mortality, and its treatment remains an unmet clinical need.^18^

Despite the broad range of topics they cover, these articles represent only a snapshot of the full spectrum of research that NIAAA-funded investigators have conducted over the last 50 years and will continue to explore in the coming decades (https://www.niaaa.nih.gov/).
